# The Tenements of New York

**Published:** 1900-03-10

**Authors:** 


### THE TENEMENTS OF NEW YORK.

There is no doubt that slumming is disagreeable work, but there is no other way if one would obtain a clear sight into the manner in which the poor live. Hence it happens that a very large number of people, who, if only they knew, would be filled with sympathy and with a desire to improve the conditions under which many of their fellow-beings are constrained to pass their lives. remain indifferent to the whole problem. Not knowing they do not care. Believing then that the indifference felt by many is merely a measure of their want of knowledge, and recognising to the full how difficult knowledge on such a subject is to obtain, the New York Charity Organisation Society has arranged an exhibition of models, plans, photographs, maps, charts, and tables of statistics dealing with the whole subject of tenement house life and sanitation. By a visit to such an exhibition it is possible to obtain in some degree that knowledge of the details of this subject which have hitherto been a closed book to all except those who, either from the nature of their work or from the strength of their sense of duty, have obtained a practical acquaintance with slum life. The importance of the subject has of late forced itself strongly upon the people of New York, for with them the housing of the working classes is almost a greater difficulty, if such be possible, than it is with us. We have to deal with a vast mass of insanitary property which has come down to us from the past, and which we cannot make up our minds to declare "unfit for human habitation," but our bye-laws may, it is to be hoped, be trusted to prevent new insanitary slums growing up around us. In New York, however, according to a statement made in the *Medical News*, it appears that new tenement houses of the most objectionable type are being erected at the rate of 2,000 a year, and it is felt that it is only by educating public opinion as to the seriousness of the evil that there is any hope of being able to obtain legal restrictions which shall render a continuance of such proceedings impossible.

